# AI‐coded radiation therapy software requires unique quality assurance

**DOI:** 10.1002/acm2.70848

**Published:** 2026-10-14

**Authors:** Alon Witztum, Yi Wang, Dongxu Wang

**Affiliations:** ^1^ Department of Radiation Oncology University of California San Francisco San Francisco California USA; ^2^ Department of Radiation Oncology Massachusetts General Hospital and Harvard Medical School Boston Massachusetts USA; ^3^ Memorial Sloan Kettering Cancer Center New York New York USA

## 1 INTRODUCTION

Software is an inseparable part of modern radiation therapy (RT), running everything from treatment planning and record‐and‐verify systems to the small scripts that physicists write to bridge gaps between clinical systems. Increasingly, software codes are crafted with the help of artificial intelligence (AI): large language models (LLMs) can now generate, modify, test, and document the software on which patient care depends. This prompts a question for our profession, known for its focus on quality assurance (QA) and safety: does software written with AI assistance demand a QA strategy of its own, distinct from the checks we already apply to any clinical software? This question spans the full range of software encountered in the clinic, from FDA‐cleared commercial products where development QA rests with the manufacturer, to in‐house scripts and tools where the physicist is often both the developer and the end user. The question is not whether such software must be verified and validated (it must), but whether AI's involvement in writing the code changes what that verification should look like, and by whom it should be performed, if it is beyond what we already require for any clinical software. To examine this emerging question, we invited two medical physicists to argue the opposing sides of the proposition: “*AI‐coded radiation therapy software requires unique* *QA*.” Arguing for the proposition is Alon Witztum, DPhil, DABR; arguing against the proposition is Yi Wang, PhD, DABR.

Dr. Alon Witztum is a medical physicist and Associate Professor in the Department of Radiation Oncology at the University of California, San Francisco (UCSF). He earned his DPhil in Oncology from the University of Oxford, completed his clinical residency in medical physics at UCSF, and is board‐certified by the American Board of Radiology. His work centers on data‐driven innovation, clinical automation, and toxicity and outcome modeling. In his position as Director of Radiation Oncology Reporting & Analytics at UCSF he develops informatics pipelines and tools for the clinic, research, quality improvement, and operations. He has contributed to textbooks and given invited talks on the use of AI in radiotherapy QA. As part of the AAPM he serves on the Big Data Subcommittee and the working group on MPPG 20: patient‐specific QA for dynamic and intensity modulated RT.

Dr. Yi Wang is a medical physicist from the Department of Radiation Oncology at Massachusetts General Hospital (MGH) and an Assistant Professor of Radiation Oncology at Harvard Medical School (HMS) in Boston, MA. He is certified in Therapeutic Radiologic Physics by the American Board of Radiology (ABR). He earned his Ph.D. in Biomedical Engineering from the University of Michigan in 2009 and completed the Harvard Medical Physics Residency Program in 2012. Since then, he has been working as a faculty physicist in the Department of Radiation Oncology at MGH. Locally, he leads an AI Lab and serves as the Director of the Mass General Brigham (MGB) Undergraduate Fellowship Program in Medical Physics. As an internationally recognized expert on AI for RT, he serves in multiple AI‐related committees, task groups, and working groups in the American Association of Physicists in Medicine (AAPM), including the Machine Intelligence Subcommittee, Ad Hoc Advisory Committee on Artificial Intelligence Boot Camps (AHAIBC), the Vice Chair of Task Group 384 (Clinical Implementation of Automated Segmentation for Adaptive Radiation Therapy), the Chair of the Working Group on Generative Artificial Intelligence (WGGenAI), and the Chair of Task Group 477 (Harmonizing Terminology of Generative Artificial Intelligence in Radiation Oncology and Radiology). He authored over 40 articles and book chapters and edited two scientific books on AI. He serves as an Associate Section Editor and sits on the Editorial Board for Practical Radiation Oncology (PRO).

## 2 OPENING STATEMENTS

### 2.1 Dr. Alon Witztum, for the proposition

Our field of RT has long depended on software. We trust treatment planning systems, record & verify systems, QA software, and countless in‐house utilities, to name just a few. We also know that trust in software is never absolute; it is earned through commissioning, QA, and independent checks.

The crux of this proposition is whether the QA we already perform on our software is sufficient regardless of how, or by whom or what, the code was developed. More precisely, it is not whether AI‐coded RT software should be trusted less than human‐written software, but whether the use of AI to generate, modify, or maintain code introduces failure modes sufficiently different to require a distinct QA strategy. I argue that it does.

The most basic objection to the proposition is simple: we usually do not know the individual developer who wrote our clinical software. If we already trust an unknown human programmer, why should an AI‐assisted programmer be treated differently? The response to this objection is that our QA is not based on knowing the programmer; it is based on understanding the development process. A human developer may make mistakes, but those mistakes usually arise within an accountable and reliable software engineering workflow that includes requirements, design, implementation, review, testing, release notes, and maintenance. AI‐generated code can appear syntactically correct and clinically plausible while reflecting no durable understanding of the clinical task, no memory of design intent, and no inherent accountability for edge cases. The risk is not that AI writes “bad code” more often than humans, but that AI can write convincing code whose failure modes are harder to anticipate and trace.

This distinction matters because our software often lives at the boundary between data handling and clinical decision‐making. Software that reformats DICOM objects, extracts plan parameters, evaluates dose‐volume metrics, or transfers information between systems may not directly deliver radiation, but it can still influence clinical judgment. Any minor array indexing error, coordinate convention mismatch, unit conversion mistake, structure‐name assumption, or silent failure can propagate throughout our treatment planning workflows.

AI‐coded software requires unique QA because the mechanism of code generation is different. LLMs are optimized to produce likely text, not verified clinical logic. They are trained across an enormous domain of human language and knowledge, and this breadth is both their strength and their weakness. Without highly specific prompting and careful review, an LLM can be pulled toward adjacent, irrelevant, or superficially similar concepts. A human developer can also misunderstand a task, but that misunderstanding is usually constrained by the envelope of their own knowledge and experience. An LLM, by contrast, may confidently blend correct clinical concepts, sophisticated software patterns, and incorrect assumptions into code that looks polished and produces plausible, but incorrect results.

This is particularly concerning when AI is used not only to write the code, but also to test it. AI is increasingly promoted as a way to “automate the boring stuff”, including unit tests, functionality checks, and documentation. This may be genuinely useful to the developer, but AI checking AI is not a substitute for independent validation. A poorly worded prompt that produces flawed code may also produce a unit test designed around the same flawed assumptions. In that scenario, rather than challenging the software, the test reinforces the original error.

We must still remain reasonable, though, and this does not mean every AI‐assisted code snippet requires a full commissioning process. The QA should be risk‐based, as it is elsewhere in our practice. But AI involvement should trigger additional questions: What parts of the code were AI‐generated? What prompts, models, and versions were used? Was the output independently reviewed by someone with domain expertise? Were tests written before or after the code? Were edge cases deliberately included? Was the code evaluated on representative local data?

The distinction between vendor and in‐house software is also important, but the structural problem is the same. For vendor products, even FDA‐cleared software, the end user may not know whether AI contributed to the code. That does not make the issue irrelevant, but it can shift much of the responsibility upstream. For in‐house code, where physicists are increasingly using AI assistants to build tools that connect directly to our clinical systems, the responsibility is more direct.

Edge cases also deserve special attention. AI‐coded software may perform well on common cases and fail silently on these exceptions. Therefore, unique QA should include adversarial testing: deliberately constructed cases that challenge assumptions about elements such as coordinate systems, dose units, structure names, missing data, and workflow interruptions.

We should also recognize that we are still in the infancy of AI‐coded software development and as a field, we have not yet determined a standardized way to safely use this technology. That uncertainty does not mean we should avoid AI‐coded software, but that we should approach it with the same caution that accompanied other major technological transitions in RT. In those cases, we built commissioning methods, QA programs, failure analyses, and professional guidance around them.

Over time our frameworks for AI‐coded software will mature, and perhaps vendors will provide robust test suites and standardized validation reports. Perhaps professional societies will define clear expectations for prompt documentation, independent code review, and clinical software risk classification. But until that confidence and infrastructure exist, we must concede that AI‐coded software requires unique QA.

Fundamentally, part of our role as medical physicists in the clinic is the safe implementation of new technology. Unique QA is not a rejection of AI‐coded software; it is the mechanism by which we can develop and deploy this software safely in clinical RT.

### 2.2 Dr. Yi Wang, against the proposition

RT software developed with AI assistance does not need additional, unique QA beyond what is already required of any clinical software. Existing regulatory frameworks already govern the safety of medical software regardless of how it was written. AI coding tools produce code that is functionally indistinguishable from human‐generated code in terms of its observable behavior and testable properties. And the practical realities of proprietary software mean that even if bespoke AI‐specific QA were desirable in principle, it would be neither feasible nor meaningful in practice. Imposing additional burdens on clinical users would generate process overhead without commensurate patient safety benefit. In the U.S., many commercial RT software products meet the definition of a medical device and are subject to FDA oversight, with applicable premarket requirements depending on device classification and regulatory pathway. The regulatory pathway is agnostic to the software's development method. The FDA's 2025 draft guidance on AI‐enabled device software functions explicitly addresses the full software lifecycle, including validation, testing, and postmarket monitoring, without carving out special rules based on whether an AI coding tool participated in development^1^, ^2^. The governing international standard, IEC 62304, provides a well‐recognized framework for software lifecycle processes, requiring analysis, architectural design, unit testing, integration testing, and risk management for all medical device software, regardless of the programming method used^3^. A manufacturer using GitHub Copilot to assist in writing device code is subject to the same verification and validation obligations as one whose developers typed every line manually. The FDA and its international counterparts are the appropriate bodies to evaluate whether a software product meets safety requirements for market release. It is their institutional responsibility, not ours as clinical users.

To understand why AI‐coded software does not warrant unique QA treatment, one must appreciate what AI coding tools are. LLMs used for code generation (e.g., those underlying GitHub Copilot) are trained on billions of lines of code written by human programmers^4^. Their output reflects the statistical patterns of their training corpus: the same conventions, idioms, and architectural decisions that experienced human developers employ. Comparative studies confirm that leading LLMs now perform at or above the level of average human programmers on standardized coding benchmarks^5^. The generated code is not alien, but a distillation of human programming practice at scale. One might argue that AI introduces novel bug patterns, but research examining code quality under AI assistance shows improvements in readability, reliability, and build success rates when AI tools are used alongside human review^6^. Characterizing AI‐generated code as categorically different from human‐generated code and therefore deserving of special scrutiny is not supported by empirical evidence.

AI‐assisted coding has undergone a real‐world safety stress test, whether the clinical community has noticed or not. As of 2025, approximately 41% of all codes written globally were AI‐generated or AI‐assisted, and roughly 62% of professional developers use AI coding tools regularly^7^. As of 2025, GitHub Copilot surpassed 20 million cumulative users and was being used by approximately 90% of Fortune 100 companies^8^. Commercial medical software, including RT products, is developed by large organizations that have enthusiastically adopted these tools to improve productivity. Clinical users have no way of knowing, and vendors are not required to disclose, what fraction of any given software release was drafted with AI assistance. Yet there is no epidemiological signal of elevated safety incidents attributable to AI‐assisted coding in medical software since these tools became widely available. If AI‐coded software were uniquely hazardous in ways that existing QA fails to catch, one would expect to see it. However, we do not.

Even setting aside the arguments above, a call for unique AI‐specific QA places an impossible burden on clinical medical physicists. Commercial RT software is proprietary. Clinical users have no access to source code, no visibility in software architecture, and no knowledge of which components, if any, were written with AI assistance. It is not possible to design a targeted QA test for a process one cannot observe. Furthermore, even if source code were available, the expertise required to evaluate the quality of AI‐generated code lies outside the training of virtually all medical physicists. One cannot reasonably propose that radiation oncology clinics outsource this evaluation to another AI tool, as doing so would simply add a layer of automated review with minimal additional human oversight, replicating the problem rather than solving it.

RT software requires rigorous acceptance testing, commissioning and ongoing QA of its functionality. The coding methods used during development didn't create additional obligations on functionality QA for the clinical end user. The responsibility for assuring that software meets engineering quality standards, regardless of whether those standards were met by a human programmer, an AI tool, or a combination of both, lies with the manufacturer and the regulatory agencies that review their work before authorizing market release.

The proposition conflates two distinct questions: (1) whether AI‐coded software must be safe (the answer is yes, and existing frameworks enforce this) and (2) whether clinical users need a new QA protocol to verify the coding process itself (the answer is no, and they have neither the access nor the expertise to do so). Medical physicists should resist the temptation to respond to new technologies by layering on new process requirements that are untethered from demonstrated safety gaps.

## 3 REBUTTALS

### 3.1 Dr. Alon Witztum, for the proposition

There are several points brought up by Dr. Wang on which we agree. AI‐generated code does reflect the patterns of the human code it learned from, regulators do bear primary responsibility for vendor products, and clinical physicists generally cannot access proprietary source code or audit a vendor's development pipeline. However, my opponent in this debate has built an argument that covers only one category of software (commercial) while not adequately covering the category growing fastest in our clinics (in‐house).

That AI code resembles human code on average tells us little about the cases that matter. The claim that AI introduces no novel failure patterns is not supported by the evidence. A 2025 analysis of more than half a million LLM‐generated code samples found that roughly one in five recommended software packages simply did not exist^9^. The researchers named this a novel class of failure, in direct contradiction of the claim that AI merely distills existing human practice. A package that does not exist is, admittedly, a basic error a developer or automated checks may catch, but the same generative mechanism can also invent plausible‐looking unit conversions or coordinate conventions that may warrant the unique QA tests we are debating here.

Dr. Wang's case rests on two pillars: a manufacturer who is legally accountable, and a regulator who reviews their work. Neither of these pillars exists for in‐house software. When a physicist uses an AI assistant to write a script that parses dose‐volume data or flags plan errors, there is no 510(k), no IEC 62304 lifecycle, no manufacturer, and no FDA review. The physicist is the developer, the integrator, and the end user at once. This is the flaw in Dr. Wang's claim that QA is “their institutional responsibility, not ours” since for in‐house code there is no them; there is only us. As AI coding assistants lower the barrier to software creation, the volume of AI‐authored code touching our systems is likely to rise. For every one of these tools, the question of appropriate QA is not a matter for the FDA; it is a matter for the person who wrote it and the clinic in which it is deployed. My opponent's argument does not reach this software at all. The division of responsibility is clear. When we purchase an FDA‐cleared product, whatever unique QA its AI‐assisted development warrants is the vendor's obligation to perform, though as I address next nothing today compels them to perform it or to disclose whether it was needed. When we write the software ourselves, that obligation is unambiguously ours.

I would gently correct a misreading on the regulatory pillar itself. The FDA's 2025 draft guidance governs AI‐enabled device software functions, meaning devices whose clinical function is performed by AI, not devices containing code that merely happens to have been authored with an AI assistant. That the guidance “carves out no special rules” for AI‐assisted coding is true precisely because it never contemplated the question. The same applies to IEC 62304, whose lifecycle processes were not written with AI‐authored code provenance specifically in mind. Whether those processes remain sufficient is not a settled premise but is the very question this debate exists to resolve.

Dr. Wang argues the absence of an epidemiological safety signal, yet he himself notes that vendors are not required to disclose AI involvement and that clinical users have no way of knowing it. We therefore have no surveillance system that tags incidents by code provenance and no mandatory reporting linked to such tagging. Additionally, these tools reached development scale only in the past two to three years, far too short a period to constitute a safety record.

Most tellingly, Dr. Wang concedes more than he intends. He dismisses using AI to review AI‐generated code because it would “simply add a layer of automated review with minimal additional human oversight, replicating the problem rather than solving it.” I agree. But that concession admits that AI‐written code can contain errors that automated review will miss. And if that is true of a checking tool, it is also true of the primary software itself. A human author holds the code's intent and can be questioned about it, but an AI author cannot. Our existing QA and review were designed around the failures humans make. Whether it also catches the different failures AI makes is exactly the gap that we must close with unique QA tests.

Finally, my opponent charges that the proposition conflates whether software must be safe with whether users must verify the coding process itself. This mischaracterizes my position. I have never asked clinical physicists to audit vendor source code. I have argued that AI authorship shifts the distribution of failures, and that this shift changes how we design the functional QA we already own as part of our clinical practice. None of this requires reading a vendor's source. As for the demand that we add no process “untethered from demonstrated safety gaps,” this is a call to invert the logic of our profession, under which TG‐100 teaches prospective risk analysis so that we anticipate failures before they reach a patient. To wait for a demonstrated safety gap before adapting our QA would reverse that logic precisely, making the patient the test case. AI‐coded software is powerful and worth embracing, and unique QA is not a barrier to that embrace but the means of achieving it safely.

### 3.2 Dr. Yi Wang, against the proposition

My opponent makes a thoughtful case, but it rests on a conflation of two distinct responsibilities and two distinct objects of scrutiny. Once those distinctions are made clear, the proposition's logic does not hold.

First, the proposition mistakes the mechanism of harm for the target of QA. The proponent argues that AI‐generated code may produce “convincing code whose failure modes are harder to anticipate and trace”. Even if we grant this characterization, clinical users are not the appropriate parties to address the coding issues due to lack of programming expertise and access to the source codes. The proponent described the failure mode as plausible but incorrect logic arising from a probabilistic language model. This is a production‐stage engineering problem, which should have been properly addressed through requirements analysis, design review, and verification testing governed by IEC 62304 and FDA guidance, all of which apply to the manufacturer before the product ever reaches a clinic. The proponent's list of special questions (e.g., “What parts of the code were AI‐generated?”, “What prompts, models, and versions were used?”) are, in fact, a developer‐side software quality checklist, not a clinical commissioning protocol. The answers to most of these questions are proprietary information for the manufacturer. While the regulatory frameworks require the manufacturers to address many of the underlying concerns during production, very few manufacturers, if any, would publicly release these critical trade secrets. Even for open‐source software, very few clinical physicists have the expertise or bandwidth to read or test tens of thousands of lines of codes in programming language that they might have never worked on before. The proponent is significantly raising the bar for clinical physicists on software commissioning, which is neither practical nor necessary.

Second, the “AI checking AI” concern applies equally to human‐written code. The proponent warns that an LLM may produce both flawed code and a flawed test suite reflecting the same misunderstanding, leaving errors undetected. This is a genuine concern in software engineering, but it describes any situation in which code and tests are written by the same author using the same assumptions, human or AI. The established defense against this problem is independent validation: a separate engineer, a separate test team, or formal verification methods. IEC 62304 addresses software system testing, including establishing tests for software requirements (5.7.1), verifying system testing (5.7.4), and maintaining system‐test records (5.7.5).^3^ FDA's General Principles of Software Validation (§4.9) additionally recommends independence of review, particularly for higher‐risk applications^10^. These established principles provide a framework for addressing correlated errors in code and tests; the possibility of such errors alone does not establish that AI‐assisted software requires a distinct clinical QA protocol. The problem is not unique to AI, and the solution already exists within the manufacturers’ development lifecycle.

Third, the empirical record warrants caution, not inaction. As I stated in the Opening Statement, there is no epidemiological signal of elevated safety incidents attributable to AI‐assisted coding in medical software since these tools became widely available. While some software engineering studies have identified higher rates of certain defects, code‐quality issues, and security vulnerabilities in AI‐generated code,^11^, ^12^ these findings do not establish that AI‐assisted software reaching clinical deployment is less safe than conventional software developed by human programmers. Two important qualifications apply. First, these studies evaluated AI‐generated code artifacts or AI‐generated contributions in software repositories rather than medical device software that had completed the full regulated development lifecycle (including independent review, verification, validation, and release testing) and therefore provide evidence only about potential code‐generation risks, not the residual defect rate of AI‐assisted software upon completion of that lifecycle. Second, software defects are not unique to AI‐assisted development. FDA recall data from 1997 to March 2024 showed that software design, likely most by human programmers before the widespread adoption of AI‐assisted coding, is the leading root cause of software‐related medical device recalls, accounting for 32% of cases^13^. The question is not whether AI‐generated code can contain defects, but whether AI‐assisted development introduces risks that cannot be adequately managed through existing verification and validation processes. If elevated defect density alone warranted unique clinical QA, we would long since have needed a unique QA protocol for human programmers, too.

Finally, the scope on in‐house developments must be correctly framed. The proponent correctly notes that in‐house tools written by clinical physicists represent a distinct governance situation where the developer and the clinical user may be the same person. This is a fair point, and it deserves a separate policy discussion. However, it does not substantiate the broad proposition that AI‐coded RT software requires unique QA, as majority of such software are FDA‐cleared commercial products distributed by vendors. A proposition must be evaluated at its stated scope. Responsibility for software quality in commercial products lies upstream, with manufacturers and regulators. Until professional societies, such as AAPM and ASTRO, define standardized validation requirements for in‐house AI‐assisted tools, clinical users should apply the same risk‐stratified acceptance testing they already use for any locally developed software, validating outputs against known benchmarks, testing edge cases, and documenting the process, in the same way as they would do for a Python script written entirely by a human.

In conclusion, the burden of proof rests with the proposition. Asserting that AI introduces novel failure modes is not the same as demonstrating that those modes escape existing regulatory and engineering controls, or that clinical end‐users are equipped to detect them when commercial vendors are not. Such demonstration has not been made. Therefore, the proposition is not necessary for safe operation of RT software, not practical given most clinical physicists’ training backgrounds, and not affordable for most, if not all, radiation oncology clinics.

## AUTHOR CONTRIBUTIONS

Dongxu Wang developed the concept and contributed to editing. Alon Witztum and Yi Wang contributed to writing and editing.

## CONFLICT OF INTEREST STATEMENT

The authors declare no conflicts of interest.
